# Cardiac–Metabolic Coupling Revealed by Lipid and Energy Metabolomics Determines 80 km Endurance Performance in Yili Horses

**DOI:** 10.3390/biology14111581

**Published:** 2025-11-12

**Authors:** Tongliang Wang, Jinlong Huang, Wanlu Ren, Jun Meng, Xinkui Yao, Hongzhong Chu, Runchen Yao, Manjun Zhai, Yaqi Zeng

**Affiliations:** 1 College of Animal Science, Xinjiang Agricultural University, Urumqi 830052, China; wtl13639911402@163.com (T.W.); 15599691616@163.com (J.H.); renwanlu@xjau.edu.cn (W.R.); junm86@xjau.edu.cn (J.M.); yaoxinkui@xjau.edu.cn (X.Y.); zhaimanjun@yeah.net (M.Z.); 2 Xinjiang Key Laboratory of Horse Breeding and Exercise Physiology, Urumqi 830052, China; 3 Horse Industry Research Institute, Xinjiang Agricultural University, Urumqi 830052, China; 4 Xinjiang Yili Kazakh Autonomous Prefecture Animal Husbandry Station, Urumqi 835000, China; 13364712998@163.com (H.C.); m18095936088@163.com (R.Y.)

**Keywords:** Yili horse, endurance, cardiac structure and function, lipidomics metabolism, energy metabolism

## Abstract

This study aimed to investigate the regulatory mechanisms underlying the relationship between cardiac structure and function and plasma metabolic characteristics in Yili horses participating in an 80-km endurance, by integrating echocardiography, lipidomics, and energy metabolomics analyses. Twenty four competing Yili horses were selected and divided based on competition outcomes: Pre-Completion Group: PCG (*n* = 6); Post-Completion Group: PoCG (*n* = 6); Overtime Completion Group: OCG (*n* = 6); and Non-Completion Group: NCG (*n* = 6). Cardiac structural and functional parameters were assessed via echocardiography, and intergroup differences were analyzed using one-way ANOVA with a significance threshold of *p* < 0.05. Plasma lipids and energy metabolites were quantified using UPLC–MS/MS, applying screening criteria of variable importance in projection (VIP) > 1, *p* < 0.05, and fold change (FC) > 1.2 or FC < 0.833. Bioinformatics analyses were subsequently conducted to identify intergroup variations and correlations. Specifically, associations between cardiac structure/function and metabolites were examined using Pearson correlation analysis, with screening criteria of *p* < 0.05 and correlation coefficient > 0.8. The results revealed the following: (1) Regarding cardiac structure and function, the PCG group exhibited significantly superior indices, including End-diastolic left ventricular diameter (LVIDd), End-diastolic left ventricular volume (EDV), stroke volume (SV), and ejection fraction (EF), compared with OCG and NCG, and LVIDd showed a highly significant negative correlation with competition completion time. (2) In metabolomic analyses, few differential metabolites were found among groups before the competition (only 60 between PCG and NCG), whereas 234 differential lipids were detected between PoCG and PCG, mainly enriched in sphingolipid metabolism and fatty acid degradation pathways. Energy metabolites showed distinct exercise-responsive patterns, with 22 differential metabolites between PCG and NCG and 21 between PoCG and PCG, significantly enriched in amino sugar and nucleotide sugar metabolism and TCA pathways. Dynamic changes in key TCA intermediates, such as citrate and succinate, reflected enhanced aerobic oxidative metabolism during endurance exercise. (3) Carnitine C18:1, Carnitine C10:2, FFA (20:3), Cer (t17:2/23:0) and 3-phenyllactic acid were significantly correlated with cardiac indicators such as LVLD and LVFWs (*p* < 0.05). In summary, performance in the 80-km endurance of Yili horses was primarily influenced by enlarged LVIDd and EDV, as well as the regulation of sphingolipid–fatty acid metabolic pathways. Triglycerides, specific acyl compounds, and ceramides may serve as potential biomarkers for evaluating endurance performance, providing a theoretical basis for scientific training and breeding of endurance horses.

## Simple Summary

Endurance places extremely high demands on the cardiac function and energy metabolism of horses. The Yili horse, as a high-quality native breed in China, performs exceptionally well in long-distance endurance events. However, the academic community still lacks a systematic understanding of the physiological regulatory mechanisms behind the 80 km endurance performance of Yili horses. This study integrates echocardiography, lipidomics, and energy metabolomics technologies to analyze Yili horses with different competition results (pre-race finishers, post-race finishers, over-time finishers, and non-finishers), systematically exploring the correlations between cardiac structural and functional characteristics, plasma lipid metabolites, energy metabolism markers, and endurance performance. The research results show that the cardiac indicators such as left ventricular end-diastolic diameter (LVIDd) and end-diastolic volume (EDV) of the group that completed the race were significantly greater than those of the group that did not complete the race, and metabolites such as triglycerides, fatty acids, and sphingomyelins are significantly associated with cardiac indicators. At the same time, pathways such as sphingolipid metabolism, fatty acid degradation, and the tricarboxylic acid cycle (TCA) are significantly activated during endurance exercise. This study not only reveals the physiological and metabolic mechanisms by which Yili horses adapt to long-distance endurance exercise, but also provides potential biomarkers and a theoretical basis for the scientific selection, training, and performance evaluation of endurance horses.

## 1. Introduction

The Fédération Equestre Internationale (FEI) describes endurance as the world’s second-largest equestrian discipline, second only to show jumping (www.fei.org accessed on 3 May 2025). The competition distances range from one-star (80 km) to three-star (160 km) levels (2023), typically designed as circuitous routes across varied terrains and completed within a single day [1]. The Yili horse is one of China’s outstanding native breeds, characterized by a relatively large body size, excellent athletic ability, and superior performance in meat and milk production, as well as strong adaptability. This breed possesses notable speed and competitive potential, along with remarkable endurance, and has achieved excellent results in numerous national competitions. It is currently one of the principal breeds used for equestrian sports in China [2].

The heart is one of the most vital organs in the body, and possessing a strong cardiac function is a fundamental prerequisite for athletic performance. Moreover, the cardiac functional demands vary across different types of athletes [3]. Young et al. [4] reported that subtle differences in training and competition can induce adaptive changes in the heart to meet the physiological demands of endurance exercise in horses. Energy metabolism refers to the biochemical processes through which nutrients such as glucose and fatty acids are converted into energy (ATP) and small-molecule metabolites. The major pathways involved include glycolysis (EMP), the tricarboxylic acid (TCA) cycle, and the pentose phosphate pathway (PPP). The role of energy metabolism in both health and disease has been increasingly recognized through its involvement in a wide range of physiological and pathological processes [5,6].

In endurance, assessing cardiac structure in combination with metabolite profiling can provide a more comprehensive and accurate reflection of the overall and dynamic physiological status of the horse [7]. Therefore, analyzing the lipid metabolome of horses can reveal specific metabolite changes, improving the scientific accuracy of predicting the athletic potential of Yili horses and filling gaps in existing research. This approach provides a theoretical basis for early selection of sport horses. Moreover, metabolomics can inform the development of tailored training programs for different competition distances, post-competition recovery strategies, and the selection of elite horses.

## 2. Materials and Methods

### 2.1. Animal Selection

Eighteen Yili horses participating in the provincial-level “Xiata Cup” 80-km endurance in Zhaosu, Xinjiang, China, were selected for this study. All horses had undergone long-term endurance training. The inclusion criteria were as follows: (1) Informed consent was obtained from the horse owners and riders; (2) Horses were confirmed to be healthy and free of lameness or any other conditions unsuitable for competition by professional veterinarians; (3) The study protocol was approved by the Xinjiang Horse Industry Association and the Animal Ethics Committee of Xinjiang Agricultural University (Approval No. 2023037; Date: 4 May 2024). All selected horses were male, and basic measurements, including body size and body weight, are presented in Table 1.

### 2.2. Study Design and Blood Sample Collection

On the day of the competition, the weather was clear, with no precipitation, temperatures ranging from 6 to 12 °C, The wind speed ranges from 0.3 m/s to 1.5 m/s, and relative humidity of 40%. The competition followed the FEI rules (https://www.fei.org, accessed on 3 May 2025). Horses were divided into four groups according to the competition outcomes: Pre-Completion Group (PCG, n = 6): referring to horses that completed the competition with qualified heart rates (Heart rate recovered to 64 bpm within 20 min), sampled before the competition; Post-Completion Group (PoCG, n = 6): elite group, referring to horses that completed the competition with qualified heart rates, sampled after the competition; Overtime Completion Group (OCG, n = 6): horses that completed the competition but were eliminated because their heart rates failed to recover to 64 bpm within 20 min; Non-Completion Group (NCG, n = 6): horses assessed as unfit to continue the competition based on their heart rate recovery, metabolic status, gait, and general condition.

Three days prior to the competition, all horses were housed in standardized stables and received uniform feeding and management. Echocardiograms were collected before the competition. Body dimension data of the experimental horses were measured using a measuring tape and a measuring rod. Body weight (BWT) was measured with a weighing scale, and body surface area (BSA, unit: m^2^) was calculated using the formula: BSA = 0.101 × BWT^2/3^.

A veterinary portable color Doppler ultrasound system (Mindray M6) with a 2.5 MHz transducer was used to acquire two sets of two-dimensional (2D) and M-mode images each, with the transducer placed between the 3rd–4th or 4th–5th right intercostal spaces of the horses. The acquired images included: B-mode right parasternal long-axis views (at end-diastole and end-systole), right parasternal left ventricular outflow tract views, and static/dynamic B-mode/M-mode right parasternal short-axis views. The maximum imaging depth was set at 30 cm, the transducer focus was fixed at 5 cm, and the maximum sector angle was 110°.

All echocardiographic examinations were performed by the same operator. Images of 3 consecutive cardiac cycles with a heart rate (HR, beats per minute, bpm) ranging from 32 to 45 bpm were obtained. Each measurement was repeated twice, and the average value was used for subsequent analyses.

Blood samples were collected both on the afternoon before the endurance and within 20 min after completion of the competition, i.e., approximately 24 h after the pre-competition sampling. This timing ensured that comparisons between pre- and post-competition results were not affected by potential circadian fluctuations in metabolism. At rest before the competition, 19 cardiac structural parameters were measured. Blood samples (10 mL) were collected from the jugular vein using sodium heparin anticoagulant tubes both at rest before each competition and immediately after completion. Samples were then centrifuged at 3500 r/min for 15 min, and plasma was transferred into cryovials, snap-frozen in liquid nitrogen, and subsequently stored at −80 °C for plasma lipidomics and energy metabolomics analyses.

### 2.3. Sample Preparation

Frozen plasma samples were removed from the −80 °C freezer and thawed on ice until no ice remained. Samples were vortexed for 10 s to mix thoroughly. An aliquot of 50 μL of each sample was transferred to a labeled centrifuge tube and 1 mL of internal standard-containing lipid extraction solution (methyl tert-butyl ether/methanol = 3:1, *v*/*v*) was added. The mixture was vortexed for 15 min, followed by the addition of 200 μL of water and vortexed for 1 min. Samples were then centrifuged at 12,000 r/min for 10 min at 4 °C. After centrifugation, 200 μL of the supernatant was transferred to a labeled centrifuge tube and dried completely. The dried residue was reconstituted with 200 μL of lipid reconstitution solution (acetonitrile/isopropanol = 1:1, *v*/*v*), vortexed for 3 min, and centrifuged at 12,000 r/min for 3 min. The resulting supernatant was collected and used for LC-MS/MS analysis.

### 2.4. Lipidomics Chromatography and Mass Spectrometry Analysis

Lipidomics analyses were performed using an Ultra Performance Liquid Chromatography system (UPLC, ExionLC™ AD; Waters, Milford, CT, USA) coupled with a Tandem Mass Spectrometer (MS/MS, QTRAP^®^ 6500+; SCIEX, Concord, Vaughan, ON, Canada).

Liquid Chromatography Conditions:

(1) Column: Thermo Accucore™ C30 column (2.6 μm, 2.1 mm × 100 mm i.d.);
(2) Mobile phases: Phase A: acetonitrile/water (60:40, *v*/*v*) containing 0.1% formic acid and 10 mM ammonium formate; Phase B: acetonitrile/isopropanol (10:90, *v*/*v*) containing 0.1% formic acid and 10 mM ammonium formate;
(3) Gradient elution program: 0 min, A/B = 80:20 (*v*/*v*); 2 min, 70:30; 4 min, 40:60; 9 min, 15:85; 14 min, 10:90; 15.5 min, 5:95; 17.3 min, 5:95; 17.5 min, 80:20; 20 min, 80:20 (*v*/*v*);
(4) Flow rate: 0.35 mL/min; column temperature: 45 °C; injection volume: 2 μL.

Mass Spectrometry Conditions:

Electrospray ionization (ESI) was employed with a source temperature of 500 °C. The mass spectrometer was operated at 5500 V in positive ion mode and −4500 V in negative ion mode. Gas 1 (GS1) was set at 45 psi, gas 2 (GS2) at 55 psi, and curtain gas (CUR) at 35 psi. In the triple quadrupole, each ion pair was scanned according to optimized declustering potential (DP) and collision energy (CE) settings.

### 2.5. Plasma Energy Metabolites

#### 2.5.1. Sample Preparation

After thawing, plasma samples were vortexed for 10 s to mix thoroughly. An aliquot of 50 μL of each sample was transferred to a pre-labeled 1.5 mL centrifuge tube, and 250 μL of 20% acetonitrile/methanol extraction solution was added. Samples were vortexed for 3 min and centrifuged at 12,000 r/min for 10 min at 4 °C.

Following centrifugation, 250 μL of the supernatant was transferred to another pre-labeled 1.5 mL centrifuge tube and stored at −20 °C for 30 min. Samples were then centrifuged again at 12,000 r/min for 10 min at 4 °C. Subsequently, 180 μL of the supernatant was passed through a protein precipitation plate and used for LC-MS/MS analysis. The processed samples were stored at −20 °C until analysis.

#### 2.5.2. Chromatography and Mass Spectrometry Acquisition Conditions

The data acquisition system mainly consisted of Ultra Performance Liquid Chromatography (UPLC, Waters ACQUITY H-Class; Waters, Milford, CT, USA) and Tandem Mass Spectrometry (MS/MS, QTRAP^®^ 6500+; SCIEX, Concord, Vaughan, ON, Canada). Liquid Chromatography Conditions: Column: ACQUITY UPLC BEH Amide column (1.7 μm, 100 mm × 2.1 mm i.d.); Mobile phases: Phase A, ultrapure water (10 mM ammonium acetate, 0.3% ammonia); Phase B, 90% acetonitrile/water (*v*/*v*); Flow rate: 0.40 mL/min; column temperature: 40 °C; injection volume: 2 μL; Gradient program: 0–1.2 min, A/B = 5:95 (*v*/*v*); 8 min, A/B = 30:70 (*v*/*v*); 9.0–11 min, A/B = 50:50 (*v*/*v*); 11.1–15 min, A/B = 5:95 (*v*/*v*).

Mass Spectrometry Conditions: ESI source temperature: 550 °C; mass spectrometer voltage: 5500 V in positive ion mode, −4500 V in negative ion mode; CUR set at 35 psi. In the QTRAP 6500+, each ion pair was scanned according to optimized DP and CE.

### 2.6. Data Analysis

Data were analyzed using SPSS 26.0. The Kolmogorov–Smirnov (K–S) test was used to assess normality. For data conforming to a normal distribution, one-way ANOVA was applied to analyze differences in cardiac structure and function among horses with different athletic performance levels. Results are presented as Mean ± SD. Identified metabolites were annotated using the KEGG, HMDB, and LIPIDMaps databases. Multivariate statistical analysis was performed using the metabolomics data processing software metaX 2.89. After data transformation, principal component analysis (PCA) and partial least squares discriminant analysis (PLS-DA) were conducted to obtain the variable importance in projection (VIP) value for each metabolite. Univariate analysis was performed using the t-test to determine the statistical significance (*p* value) of each metabolite between two groups, and fold change (FC) was calculated. The default criteria for selecting differential metabolites were VIP > 1, *p* < 0.05, and FC > 1.2 or FC < 0.833. Cluster heatmaps were generated using the R package 1.0.12 Pheatmap, and metabolite data were normalized using z-score. Bubble plots were generated using the R package ggplot2 3.5.1. Metabolic pathways were analyzed using the KEGG database; a pathway was considered enriched when x/n > y/n, and significantly enriched when *p* < 0.05.

## 3. Results and Analysis

### 3.1. Differences in Athletic Performance and Cardiac Structure and Function

Table 2 presents the cardiac structural parameters of the PCG, OCG, and NCG groups as mean ± standard deviation. As shown in the table, significant differences (*p* < 0.05) were observed between the pre-completion group and the non-completion group in several echocardiographic indices, including LVLD, LVFW, AODd, EDV, ESV, SI, LAD, LVLD, EF and SV. There were no significant differences in various indicators among other different groups” to ensure the expression is accurate.

### 3.2. PCA and OPLS Analysis

**Figure 1:**
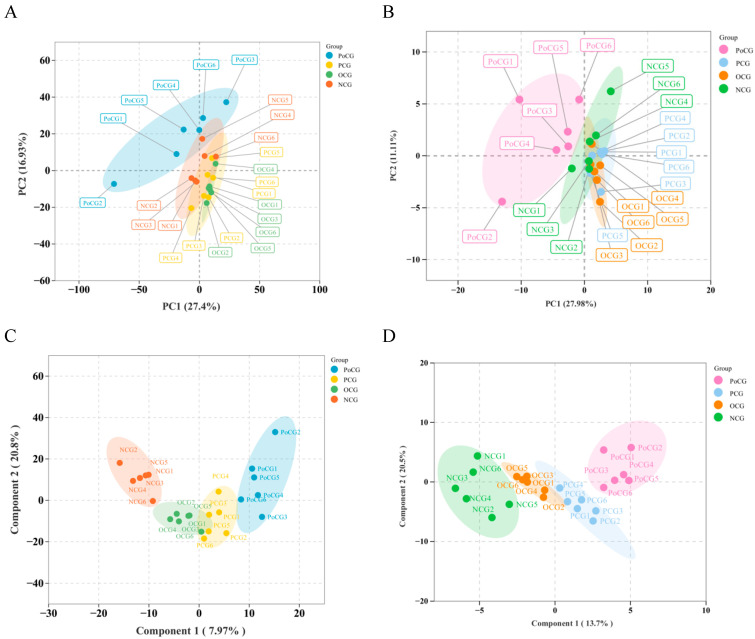
Plasma metabolomics analysis among the four groups of Yili horses. (**A**,**B**) PCA of PoCG, PCG, OCG, NCG. (**C**,**D**) OPLS-DA score plots of PoCG, PCG, OCG, NCG.

PCA and OPLS are both powerful modeling tools. PCA was performed on all samples to visually assess the overall state of the samples. In both the lipidomics and energy metabolomics datasets, the PCA results showed good clustering within each of the four groups. There was considerable overlap among groups before the competition, indicating a certain degree of homogeneity among pre-competition samples. Clear separation was observed between pre- and post-competition samples, suggesting substantial changes in metabolites between groups (Figure 1A,B). The OPLS-DA results indicated that all samples fell within the elliptical confidence interval, and distinct separation was observed between different groups. The model was stable and reliable, without overfitting, accurately describing the samples (Figure 1C,D).

### 3.3. Volcano Plots of Differential Lipid Metabolites

**Figure 2:**
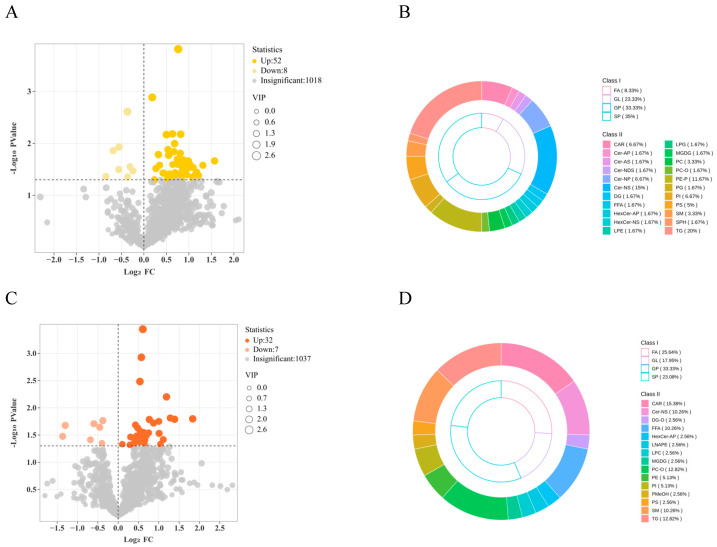

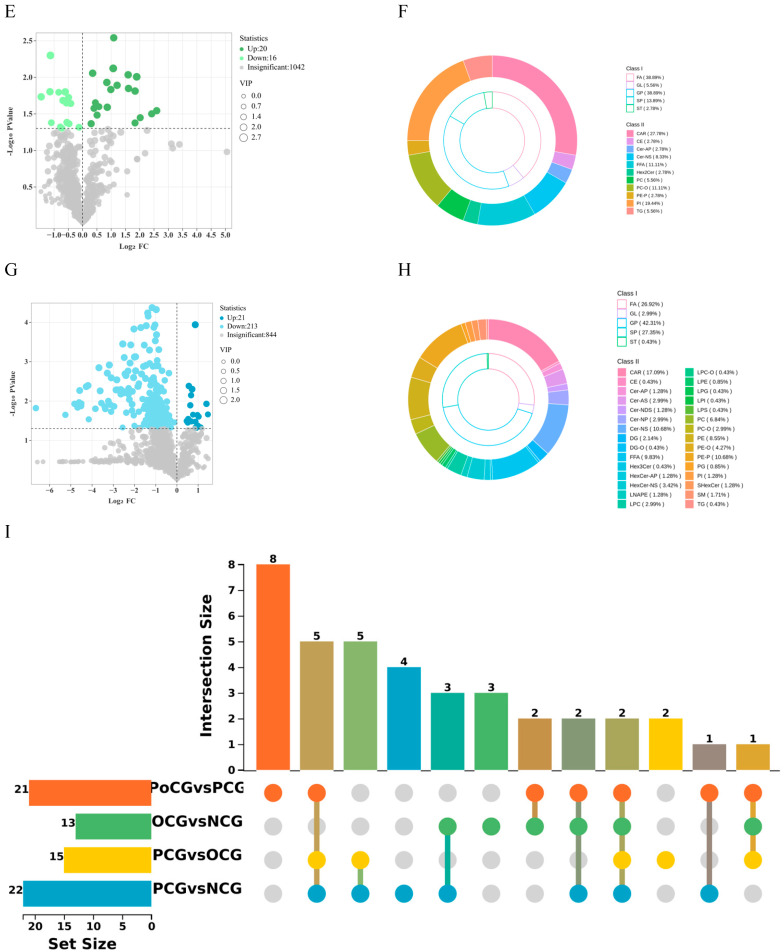
Volcano plots of lipid metabolism among groups (**A**) PCG vs. NCG; (**B**) PCG vs. NCG; (**C**) OCG vs. NCG; (**D**) OCG vs. NCG; (**E**) PCG vs. OCG; (**F**) PCG vs. OCG; (**G**) PoCG vs. PCG; (**H**) PoCG vs. PCG; (**I**) Upset Venn diagram of differential metabolites among groups in energy metabolomics. “Intersection size” represents the intersection situation of energy metabolites among different groups or between different groups. “set size” indicates the intersection situation of energy metabolites among different groups.

Differential metabolites between groups were identified using VIP > 1. Between PCG and NCG, 60 differential metabolites were detected, with 52 upregulated and 8 downregulated (Figure 2A); GLs accounted for 23.33%, GPs accounted for 33.33% (Figure 2B). Between OCG and NCG, 39 differential metabolites were detected, with 32 upregulated and 7 downregulated (Figure 2C); FAs accounted for 25.64%, GPs accounted for 33.33% (Figure 2D). Between PCG and OCG, 36 differential metabolites were detected, with 20 upregulated and 16 downregulated (Figure 2E); FAs and GPs each accounted for 38.89% (Figure 2F). Compared with the small differences observed among pre-competition groups, paired comparisons between pre- and post-competition revealed a large number of significant differential metabolites. Between PoCG and PCG, 234 differential metabolites were detected, with 21 upregulated and 213 downregulated (Figure 2G); FAs accounted for 26.92%, GPs accounted for 42.31% (Figure 2H).

Energy metabolomics showed that between PCG and NCG, 22 differential metabolites were detected, with 19 upregulated and 3 downregulated; between OCG and NCG, 15 differential metabolites were detected, with 12 upregulated and 3 downregulated; between PCG and OCG, 13 differential metabolites were detected, with 5 upregulated and 8 downregulated; between PoCG and PCG, 21 differential metabolites were detected, with 4 upregulated and 17 downregulated (Figure 2I). It is worth noting that 3-phenyllactic-acid and L-Asparagine were significantly enriched in all four groups. In the comparisons of PCG vs. NCG and OCG vs. NCG, metabolites including glutamine, alanine, glutamate, 2-oxohexanoic acid, ureidopropionic acid, adenosine triphosphate (ATP), adenosine monophosphate (AMP), adenosine diphosphate (ADP), lactic acid, and citric acid were uniquely detected in the PCG versus NCG group. This indicates that the PCG group had the highest degree of mobilization of energy substrates during endurance exercise, and its metabolic network was activated most extensively.

### 3.4. Enrichment Analysis of Differential Lipid and Energy Metabolites

To further analyze the metabolomics data, differential metabolites in each group were subjected to KEGG enrichment analysis, illustrating the potential functions of these shared or unique differential metabolites.

**Figure 3:**
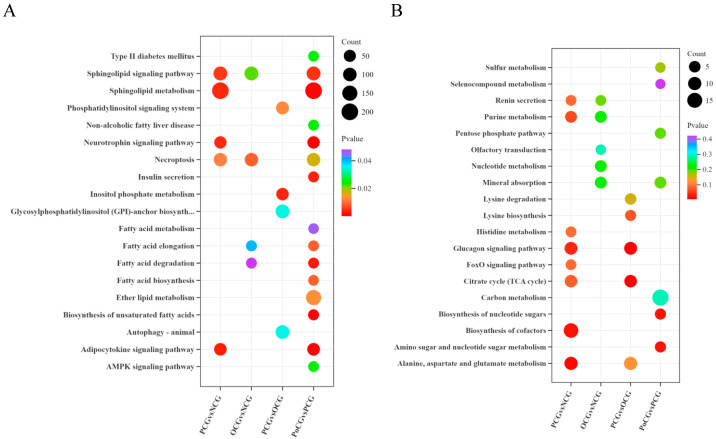
KEGG bubble chart showing the differential enrichment of metabolites between the groups. (**A**): Quantitative lipidomics; (**B**): Energy metabolomics.

Quantitative lipid metabolomics revealed that in PCG vs. NCG, the different metabolites were significantly enriched in Sphingolipid metabolism; in OCG vs. NCG, the different metabolites were significantly enriched in Necroptosis; in PCG vs. OCG, the different metabolites were significantly enriched in Inositol phosphate metabolism; and in PoCG vs. PCG, the different metabolites were significantly enriched in Sphingolipid metabolism (Figure 3A). Energy metabolomics showed that in PCG vs. NCG, the different metabolites were significantly enriched in Biosynthesis of cofactors; in OCG vs. NCG, the different metabolites were significantly enriched in Mineral absorption; in PCG vs. OCG, the different metabolites were significantly enriched in Citrate cycle (TCA cycle); and in PoCG vs. PCG, the different metabolites were significantly enriched in Amino sugar and nucleotide sugar metabolism (Figure 3B).

### 3.5. Correlation Analysis Between Core Quantitative Differential Lipid and Energy Metabolites and Key Left Ventricular Cardiac Indicators

**Figure 4:**
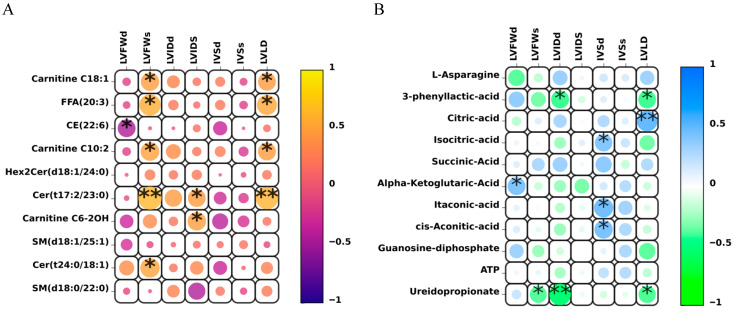
Correlation analysis of different metabolites with cardiac indicators in Yili horses. (**A**) Correlation analysis between different metabolites and plasma quantitative lipids. Yellow areas indicate a positive correlation between metabolites and cardiac indicators, with yellowish colors representing stronger positive correlations; purple areas indicate negative correlations, with darker colors representing stronger negative correlations. (**B**) Correlation analysis between different energy metabolites and cardiac indicators. Blue areas indicate a positive correlation between metabolites and cardiac indicators, with darker colors representing stronger positive correlations; green areas indicate negative correlations, with darker colors representing stronger negative correlations. Each point corresponds to a metabolite, and significance levels are indicated by * and **.

To further elucidate the potential mechanisms underlying the observed metabolic changes in Yili horses during endurance exercise, correlation analysis was performed between the significantly identified differential quantitative lipid and energy metabolites and left ventricular cardiac indicators (Figure 4). This analysis aimed to identify specific metabolites that may respond to exercise-induced adaptive changes, such as energy metabolism. Our goal was to determine potential biomarkers that may indicate changes in physiological status and adaptation to endurance exercise in horses.

The correlation analysis between lipidomic profiles and cardiac structure and function revealed several significant associations. Carnitine C6-2OH showed a significant positive correlation with LVIDs. FFA (20:3) was strongly and positively correlated with LVFWs and LVLD, and significantly correlated with LVIDs. CE (22:6) exhibited a significant negative correlation with LVFWd. Cer (t24:0/18:1) was positively correlated with LVFWs, while Cer (t17:2/23:0) showed significant positive correlations with LVFWs, LVIDs, and LVLD. In the analysis of energy metabolites, 3-phenyllactic acid displayed a significant negative correlation with LVIDd and LVLD. Isocitric acid, itaconic acid, and cis-aconitic acid were all positively correlated with IVSd. In contrast, ureidopropionate showed a significant negative correlation with LVFWs and LVLD, and an even stronger negative correlation with LVIDs.

## 4. Discussion

### 4.1. Influence of Cardiac Function on Endurance Performance

Endurance racing places high demands on equine energy metabolism, which relies on a strong cardiac pumping function for support. Long-term systematic endurance training can optimize cardiac structure and function, a process that manifests as distinct features in echocardiographic indicators and is closely related to racing performance.

From the perspective of adaptive changes in cardiac structure, long-term endurance training primarily improves pumping capacity through two pathways: ventricular chamber enlargement and adaptive thickening of the ventricular wall. Previous studies have confirmed that endurance training can induce increased left ventricular wall thickness and overall ventricular wall thickness in horses, accompanied by physiological dilation of the left ventricular cavity [8,9]. This “eccentric hypertrophy” effectively increases the left ventricular end-diastolic volume (EDV), providing a structural basis for high stroke volume (SV). For example, Sleeper et al. [10] reported in a study of 160-km endurance Arabian horses that elite horses had higher LVIDd, LVIDs, LVM, and SV compared with non-elite horses, indicating that training-induced cardiac remodeling can substantially enhance cardiac pumping efficiency.

The results of this study are consistent with these findings: the left ventricular internal diameter of horses in the completed competition group was significantly larger than that of the mid-competition elimination group, further confirming that left ventricular chamber enlargement is an important structural foundation for horses to complete long-distance endurance.

From the perspective of specialized cardiac functional adaptation, endurance athletes exhibit higher EDV, CO, EF, and FS compared with strength athletes [11]. The core mechanism lies in the fact that endurance training increases ventricular cavity volume by 15–20%, enabling a 5–6-fold increase in cardiac output [12,13,14], which meets the body’s continuous oxygen demands during prolonged exercise. In this study, LVIDd in the PCG was higher than in the NCG (*p* < 0.01). Moreover, competition completion time was negatively correlated with LVIDd, consistent with previous studies [15], suggesting that the degree of left ventricular enlargement directly affects endurance performance. A larger left ventricular cavity can accommodate more blood, increase stroke volume, reduce compensatory increases in heart rate during exercise, and delay the onset of fatigue. Sharma S et al. [16] further confirmed in a comparison among show-jumping, dressage, and endurance horses that left ventricular internal diameter is highly correlated with prolonged high-intensity endurance exercise and serves as a characteristic indicator of endurance-specific cardiac adaptation.

It is noteworthy that optimization of cardiac structure also improves the coordination of ventricular systolic and diastolic functions. For example, in human marathon runners, left and right ventricular systolic and diastolic function indicators are significantly increased, while myocardial strain in both ventricles is reduced. This change is considered a benign adaptation of the ventricles to long-term endurance exercise [17], maintaining pumping efficiency while reducing myocardial overexertion. In this study, the EDV of the elite completed competition group was higher than that of the poorer-performing group and higher than that of the ordinary group, whereas ESV was extremely significantly lower than that of the poorer-performing group and significantly lower than that of the ordinary group. The combination of increased EDV and decreased ESV forms an efficient “high diastolic reserve–low systolic residual” pattern, which not only enhances SV but also reduces ventricular diastolic load, further improving cardiac tolerance to prolonged endurance exercise. This pattern represents a core functional characteristic distinguishing elite endurance horses from ordinary horses.

### 4.2. Lipid Metabolites

Multiple studies have indicated that exercise affects systemic metabolic responses [18], and endurance training can significantly influence lipid metabolism, which plays an important role in cardiac health. The heart is a primary site for fatty acid oxidation, converting fatty acids into energy through the β-oxidation process to support myocardial cell activity. Francesca Latino et al. [19] reported that during endurance exercise, medium- and long-chain fatty acids, fatty acid oxidation products, and phospholipids undergo significant changes. Moyec et al. [20] studied 69 horses participating in national-level endurance (130–160 km) and found that protein, energy, and lipid metabolism, as well as glycoprotein content, are substantially affected by prolonged endurance exercise.

#### 4.2.1. Analysis of Differential Glycerides (GLs)

In this study, differential lipids of horses in each group were classified. Interestingly, in PCG and NCG, apart from GPs, GLs accounted for the highest proportion (GL = 23.33%), which differed from other groups, suggesting that the mobilization of GLs in elite horses may be a key factor underlying differences in athletic performance. Nosaka N [21] reported that medium-chain triglycerides (MCTs) can extend endurance time during moderate- and high-intensity exercise by increasing fat oxidation. In addition, aerobic exercise promotes the expression of adipose triglyceride lipase (ATGL) in skeletal muscle, thereby enhancing lipolysis and lipid metabolism [22]. Notably, during low-intensity exercise in endurance, the oxidation rates of fat and glucose are higher [23]. KIM J [24] demonstrated in mice that endurance training increases the content of fatty acid translocases in skeletal muscle, which is positively correlated with whole-body fat oxidation, potentially enhancing endurance performance. The importance of triglycerides (TG) stored in adipose tissue as an energy source decreases with increasing exercise intensity but increases with prolonged exercise duration. Endurance athletes can consume more intramuscular triglycerides during exercise, and endurance training increases the proportion of intramuscular triglycerides mobilized during exercise. Lipid catabolism offers an energetic advantage over carbohydrate metabolism, which is particularly crucial for sustaining endurance performance. Girona J et al. [25], using lipidomics, found that in mice with high-fat diet-induced insulin resistance, hepatic triglyceride content was positively correlated with upregulated metabolites in the myocardium, and myocardial lipid composition was significantly correlated with multiple plasma variables [26,27]. In this study, the secondary lipid classification (TG) showed significant differences between groups, with the greatest differences observed between PCG and NCG. Collectively, these prominent intergroup TG differences imply that differential remodeling of lipid metabolites—centered on TG as a core energy storage molecule—serves as a key mechanistic mediator underlying enhanced endurance performance, likely by promoting intramuscular triglyceride mobilization and optimizing lipid catabolism-dependent energy supply to meet the metabolic demands of prolonged exercise.

Comparison of lipid profiles between PCG and PoCG revealed distinct patterns compared to inter-group differences observed in the pre-competition resting state. Although glycerophospholipids (GPs) remained the most significantly altered lipid class in both comparisons, the second most affected class shifted from glycerolipids (GLs) to sphingolipids (SPs), which accounted for 27.3% of the differential lipids in the pre- vs. post-competition contrast. A study on rats before and after exercise reported that myocardial ceramide (Cer) levels decreased following exercise, returned to baseline after 90 min, and exceeded control levels at exhaustion. This fluctuation may be related to changes in peripheral insulin sensitivity.

#### 4.2.2. Analysis of Differential Fatty Acyls (FAs)

Exercise plays a crucial role in regulating FFA metabolism and enhancing cardiac function. For instance, high-intensity interval training (HIT) has been shown to improve cardiopulmonary and cardiovascular functions by enhancing the body’s ability to utilize fatty acids as an energy source [28]. This form of exercise increases the efficiency of mitochondrial oxidation, thereby helping to maintain a better energy balance and reducing oxidative stress, which is crucial for preventing diseases such as diabetic cardiomyopathy [29,30]. Carnitine serves as a candidate for fatty acid oxidation during exercise. During high-intensity exercise, glycolysis rapidly increases, producing excess acetyl-CoA in the mitochondria, which is buffered by carnitine to form acetylcarnitine [31]. Correlation analysis between FAs and cardiac structure and function revealed that Carnitine C6-2OH was significantly positively correlated with LVIDs, while Carnitine C10:2 and Carnitine C18:1 were significantly positively correlated with LVFWs and LVLD. The metabolite of ceramides, sphingosine-1-phosphate (S1P), is crucial for cardiac development and is involved in the formation of the primitive heart tube and the migration of cardiomyocytes. Therefore, the present study suggests that Carnitine C6-2OH, Carnitine C10:2, and Carnitine C18:1 may have a potential association with cardiac structure. Given that the current study has not yet clarified their specific mechanisms of action and biological significance, the validity of this association and its regulatory pathways still need to be further clarified by subsequent studies. Meienberg [32] reported that blood acetylcarnitine levels significantly increased after endurance exercise, suggesting that acetylcarnitine production may support the regulation of energy metabolism during exercise and assist in post-exercise muscle fatigue recovery. In this study, Ureidopropionate, 3-phenyllactic-acid, L-Asparagine showed significant differences between groups, indicating its association with endurance performance. Additionally, Carnitine C6-2OH, Carnitine C10:2, and Carnitine C18:1 levels differed significantly among groups in the Yili horse 80 km endurance. This may be due to the high-energy demands of skeletal muscle during intense exercise, which increase β-oxidation activity of fatty acids in muscle, leading to a rapid rise in plasma acetylcarnitine levels. These changes may indirectly influence endurance performance by affecting metabolic pathways.

#### 4.2.3. Analysis of Differential Sphingolipids (SPs)

Ceramides are the simplest sphingolipids and serve as precursors for various complex sphingolipids, constituting a fundamental class of lipids in cell membranes. In this study, Hex2Cer(d18:1/24:0) levels differed significantly among groups in the Yili horse 80 km endurance. Moreover, Cer(t24:0/18:1) was significantly positively correlated with LVFWs, and Cer(t17:2/23:0) was significantly positively correlated with LVFWs, LVIDs, and LVLD, potentially related to myocardial cell metabolism and signal transduction. These effects may influence endurance performance in horses by modulating myocardial cell metabolism and signaling. Simon N. J. [33] reported that ceramides impair myocardial contractile function by activating protein kinase C (PKC) and altering phosphorylation states of myofilament proteins, leading to reduced maximal force generation. Other studies have shown that ceramides are key contributors to the development of dilated cardiomyopathy in lipotoxic animal models [34,35].

#### 4.2.4. Analysis of Energy Metabolites

Energy metabolism refers to the processes by which living organisms obtain, convert, and utilize energy through a series of biochemical reactions. It encompasses not only energy acquisition and storage but also its release and utilization to support diverse physiological functions and activities. The primary pathway for energy metabolism is oxidative phosphorylation, which occurs in the mitochondria and generates large amounts of adenosine triphosphate (ATP) to meet cellular energy demands. Moreover, energy metabolism is closely associated with cellular growth, division, and apoptosis [36]. In the present study, significant alterations were observed in metabolites such as isocitric acid (VIP = 1.82), itaconic acid (VIP = 1.71), and cis-aconitic acid (VIP = 1.67). These changes may result from the markedly increased mitochondrial energy demand during prolonged aerobic exercise. Under high-intensity, sustained running, the activities of key enzymes—such as citrate synthase and isocitrate dehydrogenase—may undergo transient inhibition, leading to the accumulation of isocitric acid in the bloodstream. Meanwhile, fluctuations in mitochondrial membrane potential and reduced oxidative phosphorylation efficiency may further contribute to these metabolic shifts [37]. Exercise also activates macrophages in muscle and blood, inducing IRG1 expression, which catalyzes the conversion of cis-aconitate into itaconic acid. Consequently, itaconic acid cannot be readily converted into isocitric acid, resulting in its temporary accumulation [38]. Collectively, these findings suggest that extreme aerobic energy demand leads to the accumulation of upstream intermediates in the tricarboxylic acid (TCA) cycle.

### 4.3. Effects of Plasma Metabolic Mechanisms in Yili Horses

Differential lipids among the groups were commonly enriched in 18 pathways. The differential lipids between PCG and NCG were enriched in the sphingolipid signaling pathway and sphingolipid metabolism, highlighting the key roles of lipid energy mobilization and cellular signal regulation. During endurance exercise, the body requires efficient mobilization of adipose tissue; horses that completed the competition may utilize more active sphingolipid metabolism to effectively promote fatty acid oxidation for energy supply and maintain cellular homeostasis under stress, thereby supporting sustained exercise. Enrichment in the adipocytokine signaling pathway suggests that adipokines contribute to promoting lipolysis, improving glucose homeostasis, and suppressing excessive inflammatory responses during endurance racing [37,38]. Horses that completed the competition may more efficiently coordinate systemic energy distribution and mitigate exercise-induced inflammation through this pathway, thereby enhancing performance efficiency and recovery capacity.

Differential lipids between PCG and PoCG were enriched in fatty acid degradation, indicating that horses efficiently mobilized fat reserves for energy during exercise. In the later stages of endurance exercise, muscle glycogen is gradually depleted, and fatty acid oxidation becomes a critical energy source to maintain exercise intensity. Activation of this pathway implies that the body enhances mitochondrial β-oxidation, continuously converting free fatty acids (FFAs) into ATP to meet substantial energy demands. Pathways such as fatty acid biosynthesis, biosynthesis of unsaturated fatty acids, and fatty acid elongation reveal post-exercise repair and adaptation processes. This may indicate intense post-competition energy mobilization and consumption (fatty acid biosynthesis), while simultaneously initiating repair and reconstruction programs (biosynthesis of unsaturated fatty acids, fatty acid elongation). Changes in insulin secretion act as a “metabolic switch” coordinating this conversion [39]. The core biological significance lies in efficiently utilizing and precisely regulating fat-derived energy to ensure exercise performance, while timely structural lipid synthesis supports recovery and long-term adaptation.

The consistent detection of 3-phenyllactic acid (3-PLA) and L-Asparagine (L-Asn) across all four groups of the 80 km equine endurance race reflects universal metabolic/stress pathways activated by prolonged exercise. L-Asn supports nitrogen balance and energy supply, while 3-PLA exerts anti-inflammatory/antioxidant effects. Their concentration differences may relate to race outcomes. Post-race levels could indicate recovery capacity, highlighting their potential as biomarkers for endurance adaptation and fatigue. Notably, 3-phenyllactic acid exhibits a significant correlation with left ventricular internal diameter at end-diastole (LVIDd), and left ventricular end-diastolic volume (LVLD). This finding suggests a close association between ureidopropionate and cardiac structure/function as well as energy metabolism.

Sphingolipid metabolism directly affects cardiac function. As a high-energy-demand organ, the heart requires a stable energy supply, and sphingolipids play a crucial role in energy metabolism. Abnormal sphingolipid metabolism may lead to myocardial cell hypertrophy, arrhythmias, and other issues, resulting in impaired cardiac function. For example, some studies have found that increased sphingolipid content within myocardial cells is closely associated with the development of heart failure [40,41]. Research also indicates that individuals engaging in regular aerobic exercise exhibit significantly improved levels of sphingolipid metabolites in the blood, likely due to enhanced lipid metabolic efficiency induced by exercise [42]. In this study, sphingolipid metabolic pathways were enriched across different groups, indicating that sphingolipid metabolism plays an important role in both cardiac health and endurance performance.

## 5. Conclusions

This study integrates echocardiography, lipidomics, and energy metabolomics to systematically elucidate the cardiac structural–functional basis and plasma metabolic regulatory mechanisms underlying performance differences in Yili horses during an 80 km endurance. The research results show that the cardiac indicators such as left ventricular end-diastolic diameter (LVIDd) and end-diastolic volume (EDV) of the group that completed the race were significantly greater than those of the group that did not complete the race. Prior to the competition, elite horses already exhibited baseline advantages in TG, specific FAs, and ceramides. Carnitine C18:1, Carnitine C10:2, FFA(20:3), Cer(t17:2/23:0) and 3-phenyllactic acid were significantly correlated with cardiac indicators such as LVLD and LVFWs (*p* < 0.05), representing a multi-omics biomarker combination for athletic performance. In elite horses, plasma levels of citrate, succinate, and other TCA cycle intermediates were significantly elevated, accompanied by coordinated activation of the AMPK signaling pathway, fatty acid degradation, and sphingolipid metabolic pathways. In summary, combining echocardiography with plasma metabolomics allows effective assessment of equine athletic potential and provides important theoretical guidance and practical implications for precision breeding and scientific training of endurance horses.

## Figures and Tables

**Table 1:** Differences in cardiac structure and function in different exercise performance.

Indicator	Excellent Group	Ordinary Group	Poor Group
	Mean ± SD	Mean ± SD	Mean ± SD
Body weight (kg)	376.92 ± 5	381.19 ± 6.92	369.21 ± 23.17
Body surface area (m^2^)	5.25 ± 0.05	5.29 ± 0.06	5.18 ± 0.22
Width height (cm)	150.33 ± 1.97	149.83 ± 4.62	148.83 ± 3.19
Body length (cm)	153.17 ± 1.6	151 ± 4.38	151.33 ± 4.63
Thoracic circumference (cm)	168.08 ± 0.67	167.83 ± 2.99	167 ± 4.2
Circumference of cannon bone (cm)	18.78 ± 0.68	19.58 ± 0.92	18.95 ± 0.85
Chest depth (cm)	63.58 ± 1.66	62.25 ± 0.69	62.58 ± 1.66
Chest width (cm)	38.42 ± 1.53	36.42 ± 2.46	36.33 ± 2.02

**Table 2:** Structural variability of the heart for different exercise performance.

Indicator	PCG	OCG	NCG
LVFWd (cm)	2.15 ± 0.31	2.35 ± 0.38	2.31 ± 0.29
LVFWs (cm)	4.32 ± 0.21 Aa	4.01 ± 0.28 ABb	3.69 ± 0.25 Bc
LVIDd (cm)	10.56 ± 0.34 Aa	10.19 ± 0.17 ABb	10.1 ± 0.09 Bb
LVIDs (cm)	5.66 ± 0.14	5.44 ± 0.34	5.58 ± 0.25
IVSd (cm)	2.83 ± 0.39	2.72 ± 0.42	2.88 ± 0.43
IVSs (cm)	4.15 ± 0.84	4.53 ± 0.34	4.53 ± 0.4
RVDd (cm)	3 ± 0.49	2.74 ± 0.38	2.84 ± 0.83
RVDs (cm)	1.79 ± 0.28	1.76 ± 0.2	1.73 ± 0.77
LVLD (cm)	15.94 ± 0.42 Aa	15.25 ± 0.27 Bb	14.69 ± 0.48 Bc
MVD (cm)	9.75 ± 0.4	9.77 ± 0.57	9.62 ± 0.62
LADd (cm)	9.87 ± 0.49 a	9.11 ± 0.54 b	8.96 ± 0.66 b
LADs (cm)	11.46 ± 0.42 a	10.44 ± 0.59 b	11.04 ± 1.18
AODd (cm)	6.09 ± 0.33 a	5.64 ± 0.27 b	5.71 ± 0.24 b
AODs (cm)	6.89 ± 0.51	6.75 ± 0.8	6.53 ± 1.2
PADd (cm)	4.47 ± 0.38	4.95 ± 0.46	4.82 ± 0.47
PADs (cm)	5.56 ± 0.49	6.08 ± 0.35	5.62 ± 0.64
MWTd (cm)	2.49 ± 0.19	2.53 ± 0.25	2.6 ± 0.28
RWTd (cm)	0.47 ± 0.04	0.5 ± 0.05	0.51 ± 0.06
LVM (g)	2671.04 ± 285.5	2593.37 ± 367.64	2646.66 ± 394.5
Heart rate	35.29 ± 1.42	35.09 ± 3.22	38.04 ± 5.35
EDV (mL)	656.37 ± 45.08 Aa	602.18 ± 34.94 ABb	581.78 ± 45.63 Bb
ESV (mL)	186.47 ± 11.19 Aa	207.87 ± 11.49 ABb	214.76 ± 23.4 Bb
EF (%)	0.72 ± 0.02 Aa	0.65 ± 0.02 ABb	0.63 ± 0.06 Bb
ET	0.59 ± 0.02	0.58 ± 0.05	0.63 ± 0.09
SV (mL)	469.9 ± 41.34 Aa	394.31 ± 34.59 ABb	367.02 ± 59.1 Bb
SI	89.51 ± 7.68 Aa	74.59 ± 6.96 ABb	70.96 ± 11.28 Bb
CO (mL/min)	16,571.67 ± 1487.14	13,878.4 ± 2170.94	14,134.92 ± 3765.31
CI	3156.5 ± 273.12	2626.25 ± 427.28	2726.25 ± 692.57
FS%	48.43 ± 4.23	44.5 ± 1.83	44.78 ± 2.26
VTI	3.16 ± 0.27	2.63 ± 0.43	2.73 ± 0.69
LV MASS-I	509.09 ± 57.12	490.18 ± 67.96	512.7 ± 82.34

Notes: Comparison of cardiac parameters among the three groups. IVSd, end-diastolic interventricular septal thickness; LVIDd, end-diastolic left ventricular diameter; LVFWd, end-diastolic left ventricular free wall thickness; RVDs, end-systolic right ventricular diameter; IVSs, end-systolic interventricular septal thickness; LVIDs, end-systolic left ventricular diameter; LVFWs, end-systolic left ventricular free wall thickness; LVLD, left ventricle long axis diameter; MVD, mitral valve diameter; LADd, end-diastolic left atrial diameter; LADs, end-systolic left atrial diameter; AODd, end-diastolic aortic root diameter; PADd, end-diastolic pulmonary artery diameter; AODs, end-systolic aortic root diameter; PADs, end-systolic pulmonary artery diameter; EDV, end-diastolic left ventricular volume; ESV, end-systolic left ventricular volume; EF, ejection fraction; SV, stroke volume; FS, fractional shortening; LVM, left ventricular myocardial mass; HR, heart rate. Capital letters on the shoulder of the peer indicated that the difference was extremely significant (*p* < 0.01), lowercase letters indicated that the difference was significant (*p* < 0.05), and no letters indicated that there was no significant difference (*p* > 0.05).

## Data Availability

The original contributions presented in the study are included in the article, further inquiries can be directed to the corresponding author.
